# A Unified Deep Framework for Joint 3D Pose Estimation and Action Recognition from a Single RGB Camera

**DOI:** 10.3390/s20071825

**Published:** 2020-03-25

**Authors:** Huy Hieu Pham, Houssam Salmane, Louahdi Khoudour, Alain Crouzil, Sergio A. Velastin, Pablo Zegers

**Affiliations:** 1 Cerema Research Center, 31400 Toulouse, France; hieuhuy01@gmail.com (H.H.P.); louahdi.khoudour@cerema.fr (L.K.); 2 Informatics Research Institute of Toulouse (IRIT), Université de Toulouse, CNRS, 31062 Toulouse, France; alain.crouzil@irit.fr; 3 Vingroup Big Data Institute (VinBDI), Hanoi 10000, Vietnam; 4 Clay AIR, Software Solution, 33000 Bordeaux, France; psalmane@clayair.io; 5 School of Electronic Engineering and Computer Science, Queen Mary University of London, London E1 4NS, UK; 6 Zebra Technologies Corp., London SE1 9LQ, UK; 7 Department of Computer Science and Engineering, University Carlos III de Madrid, 28270 Colmenarejo, Spain; 8 Aparnix, Santiago 7550076, Chile; pablozegers@gmail.com

**Keywords:** human action recognition, 3D pose estimation, RGB sensors, deep learning

## Abstract

We present a deep learning-based multitask framework for joint 3D human pose estimation and action recognition from RGB sensors using simple cameras. The approach proceeds along two stages. In the first, a real-time 2D pose detector is run to determine the precise pixel location of important keypoints of the human body. A two-stream deep neural network is then designed and trained to map detected 2D keypoints into 3D poses. In the second stage, the Efficient Neural Architecture Search (ENAS) algorithm is deployed to find an optimal network architecture that is used for modeling the spatio-temporal evolution of the estimated 3D poses via an image-based intermediate representation and performing action recognition. Experiments on Human3.6M, MSR Action3D and SBU Kinect Interaction datasets verify the effectiveness of the proposed method on the targeted tasks. Moreover, we show that the method requires a low computational budget for training and inference. In particular, the experimental results show that by using a monocular RGB sensor, we can develop a 3D pose estimation and human action recognition approach that reaches the performance of RGB-depth sensors. This opens up many opportunities for leveraging RGB cameras (which are much cheaper than depth cameras and extensively deployed in private and public places) to build intelligent recognition systems.

## 1. Introduction

Human Action Recognition (HAR) from videos has been researched for decades, since this topic plays a key role in various areas such as intelligent surveillance, human–robot interaction, robot vision and so on. Although significant progress has been achieved in the past few years, building an accurate, fast and efficient system for the recognition of actions in unseen videos is still a challenging task due to several obstacles, e.g., changes in camera viewpoint, occlusions, background, speed of motion, etc. Traditional approaches on video-based action recognition [1] have focused on extracting hand-crafted local features and building motion descriptors from RGB sensors. Many spatio-temporal representations of human motion have been proposed and widely exploited with success such as Scale Invariant Feature Transform (SIFT) [2], Histograms of Optical Flow (HOF) [3] or Cuboids [4]. However, one of the major limitations of these approaches is the lack of 3D structure from the scene and recognizing human actions based only on RGB information is not enough to overcome the current challenges in the field.

The rapid development of depth-sensing time-of-flight sensor technology has helped in dealing with this problem, which is considered complex for traditional sensors. Low-cost and easy-to-use depth sensors can provide detailed 3D structural information of human motion. In particular, most of the current depth sensors have integrated real-time skeleton estimation and tracking frameworks [5], facilitating the collection of skeletal data. This is a high-level representation of the human body, which is suitable for the problem of motion analysis. Hence, exploiting skeletal data for 3D action recognition opens up opportunities for addressing the limitations of RGB-based solutions and many skeleton-based action recognition approaches have been proposed [6,7,8,9,10]. However, depth sensors have some significant drawbacks with respect to 3D pose estimation. For instance, they are only able to operate up to a limited distance and within a limited field of view. Moreover, a major drawback of low-cost depth sensors is their inability to work in bright light, especially sunlight [11].

**Figure 1:**
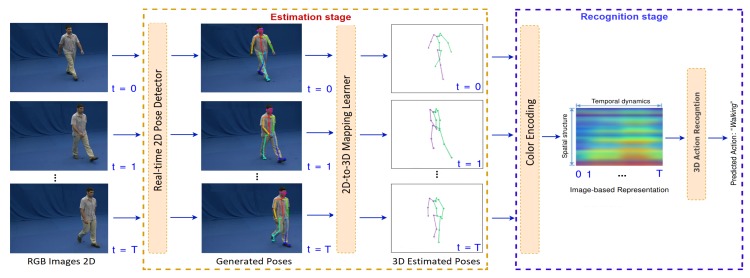
Overview of the proposed method. In the estimation stage, a real-time multi-person 2D pose detector, such as OpenPose [12] or AlphaPose [13], is used to generate 2D human body keypoints. A deep neural network is then trained to produce 3D poses from the 2D detections. In the recognition stage, the 3D estimated poses are encoded into a compact image-based representation and finally fed into a deep convolutional network for supervised classification task, which is automatically searched by the ENAS algorithm [14].

The focus in this paper is therefore to propose a 3D skeleton-based action recognition approach without the need for depth sensors. Specifically, we are interested in building a unified deep framework for both 3D pose estimation and action recognition from RGB video sequences provided by single color sensors. As shown in Figure 1, the approach consists of two stages. In the first, estimation stage, the system recovers the 3D human poses from the input RGB video. In the second, recognition stage, an action recognition approach is developed and stacked on top of the 3D pose estimator in a unified framework, where the estimated 3D poses are used as inputs to learn the spatio-temporal motion features and predict action labels.

There are four hypotheses that motivate us to build a deep learning framework for human action recognition from 3D poses. First, actions can be correctly represented through 3D pose movements [15,16]. Second, the 3D human pose has a high-level of abstraction with much less complexity compared to RGB and depth streams. This makes the training and inference processes much simpler and faster. Third, depth sensors can provide highly accurate skeletal data for 3D action recognition. However, they are expensive and not always available (e.g., for outdoor scenes). A fast and accurate approach of 3D pose estimation from only RGB input is highly desirable. Fourth, state-of-the-art 2D pose detectors [12,13,17] are able to provide 2D poses with a high degree of accuracy in real time. Meanwhile, deep networks have proved their capacity to learn complex functions from high-dimensional data. Hence, a simple network model can also learn a mapping to convert 2D poses into 3D. The effectiveness of the proposed method is evaluated on public benchmark datasets (Human3.6M [18] for 3D pose estimation and MSR Action3D [19] and SBU [20] for action recognition). Beyond the initial expectations, the experimental results demonstrate state-of-the-art performance on the targeted tasks (Section 4.3) and support the hypotheses above. Furthermore, we show that this approach has a low computational cost (Section 4.4). Overall, our main contributions are as follows:

- First, we present a two-stream, lightweight neural network to recover 3D human poses from RGB images provided by a monocular camera. The proposed method achieves state-of-the-art result on 3D human pose estimation task and benefits action recognition. The novelty of the study is that a very simple deep neural network could be trained effectively to learn a 2D-to-3D mapping for the task of 3D human estimation from color sensors.
- Second, we propose to put an action recognition approach on top of the 3D pose estimator to form a unified framework for 3D pose-based action recognition. It takes the 3D estimated poses as inputs, encodes them into a compact image-based representation and finally feeds to a deep convolutional network, which is designed automatically by using a neural architecture search algorithm. Surprisingly, the experiments show that we reached state-of-the-art results on this task, even when compared with methods using depth cameras.

The rest of this paper is organized as follows. A review of the related work is presented in Section 2. The proposed method is explained in Section 3. Experiments are provided in Section 4 and Section 5 concludes the paper.

## 2. Related Work

This section reviews two main topics that are directly related to the proposed approach, i.e., 3D pose estimation from RGB images and 3D pose-based action recognition. An extensive literature review is beyond the scope of this section. Instead, the interested reader is referred to the surveys of Sarafianos et al. [21] for recent advances in 3D human pose estimation and Presti et al. [22] for 3D skeleton-based action recognition.

### 2.1. 3D Human Pose Estimation

The problem of 3D human pose estimation has been intensively studied in recent years. Almost all early approaches for this task were based on feature engineering [18,23,24], while the current state-of-the-art methods are based on deep neural networks [25,26,27,28,29,30]. Many of them are regression-based approaches that directly predict 3D poses from RGB images via 2D/3D heatmaps. For instance, Li et al. [25] designed a deep convolutional network for human detection and pose regression. The regression network learns to predict 3D poses from single images using the output of a body part detection network. Tekin et al. [26] proposed to use a deep network to learn a regression mapping that directly estimates the 3D pose in a given frame of a sequence from a spatio-temporal volume centered on it. Pavlakos et al. [27] used multiple fully convolutional networks to construct a volumetric stacked hourglass architecture, which can recover 3D poses from RGB images. Pavllo et al. [28] exploited a temporal dilated convolutional network [31] for estimating 3D poses. However, this approach led to a significant increase in the number of parameters as well as the required memory. Mehta et al. [29] introduced a real-time approach to predict 3D poses from a single RGB sensor. They used ResNets [32] to jointly predict 2D and 3D heatmaps as regression tasks. Recently, Katircioglu et al. [30] introduced a deep regression network for predicting 3D human poses from monocular images via 2D joint location heatmaps. This architecture is in fact an overcomplete autoencoder that learns a high-dimensional latent pose representation and accounts for joint dependencies, in which a Long Short-Term Memory (LSTM) network [33] is used to enforce temporal consistency on 3D pose predictions.

To the best of our knowledge, several studies [27,29,30] stated that regressing the 3D pose from 2D joint locations is difficult and not too accurate. However, motivated by Martinez et al. [34], we believe that a simple neural network can learn effectively a direct 2D-to-3D mapping. Therefore, this paper aims at proposing a simple, effective and real-time approach for 3D human pose estimation that benefits action recognition. To this end, a two-stream deep neural network that performs 3D pose predictions from the 2D human poses is designed and optimized. These 2D poses are generated by a state-of-the-art 2D detector, which can run in real time for multiple people. We empirically show that although the proposed approach is computationally inexpensive, it is still able to improve the state-of-the-art.

### 2.2. 3D Pose-Based Action Recognition

Human action recognition from skeletal data or 3D poses is a challenging task. The methods used in previous works on this topic can be divided into two main groups. The first group [6,9,35] extracts hand-crafted features and uses probabilistic graphical models, e.g., Hidden Markov Model (HMM) [35] or Conditional Random Field (CRF) [36] to recognize actions. However, almost all these approaches require a lot of feature engineering. The second group [37,38,39] considers the 3D pose-based action recognition as a time-series problem and proposes to use Recurrent Neural Networks with Long Short-Term Memory units (RNN-LSTMs) [33] for modeling the dynamics of the skeletons. Although RNN-LSTMs are able to model the long-term temporal characteristics of motion and have advanced the state-of-the-art, this approach feeds raw 3D poses directly into the network and just considers them as a kind of low-level feature. The large number of input features makes RNNs very complex and may easily lead to overfitting. Moreover, many RNN-LSTMs act merely as classifiers and cannot extract high-level features for recognition tasks [40].

In the literature, 3D human pose estimation and action recognition are closely related. However, both problems are generally considered to be two distinct tasks [41]. Although some approaches have been proposed for tackling the problem of jointly predicting 3D poses and recognizing actions in RGB images or video sequences [42,43,44], they are data-dependent and require a lot of feature engineering, except the work of Luvizon et al. [44]. Unlike previous studies, a multitask learning framework for 3D pose-based action recognition is proposed here by reconstructing 3D skeletons from RGB images and exploiting them for action recognition in a joint way. Experimental results on public and challenging datasets show that the framework can solve the two tasks in an effective way.

## 3. Proposed Method

This section presents the proposed method. First, the approach for 3D human pose estimation is presented, followed by the proposed solution for 3D pose-based action recognition.

### 3.1. Problem Definition

Given an RGB video clip of a person who starts to perform an action at time t=0 and ends at t=T, the problem studied in this work is to generate a sequence of 3D poses $P=(p_{0},\ldots ,p_{T})$, where $p_{i}\in R^{3\times M}$, i∈{0,…,T} at the estimation stage, in which *M* denotes the number of keypoints for the pose $p_{i}$. The generated P is then used as input for the recognition stage to predict the corresponding action label A by a supervised learning model. See Figure 1 for an illustration of the problem.

### 3.2. 3D Human Pose Estimation

Given an input RGB image $I\in R^{W\times H\times 3}$, we aim to estimate the body joint locations in the 3-dimensional space, noted as ${\hat{p}}_{3D}$ $\in R^{3\times M}$. To this end, we first run any state-of-the-art human 2D pose detector, in this case OpenPose [12], to produce a series of 2D keypoints $p_{2D}\in R^{2\times N}$. To recover the 3D joint locations, we try to learn a direct 2D-to-3D mapping $f_{r}$: $p_{2D}\overset{f_{r}}{\mapsto }{\hat{p}}_{3D}$. This transformation can be implemented by a deep neural network in a supervised manner

(1) $${\hat{p}}_{3D}=f_{r}\left(p_{2D},\theta \right),$$

where θ is a set of trainable parameters of the function $f_{r}$. To optimize $f_{r}$, the prediction error is minimized over a labelled dataset of C poses by solving the optimization problem

(2) $$\underset{\theta }{arg min}\;\frac{1}{C}\sum_{n=1}^{C}L\left(f_{r}\left(x_{i}\right),y_{i}\right).$$

Here $x_{i}$ and $y_{i}$ are the input 2D poses and the ground truth 3D poses, respectively; L denotes a loss function. Here, the robust Huber loss [45] is used to deal with outliers.

#### Network Design

**Figure 2:**
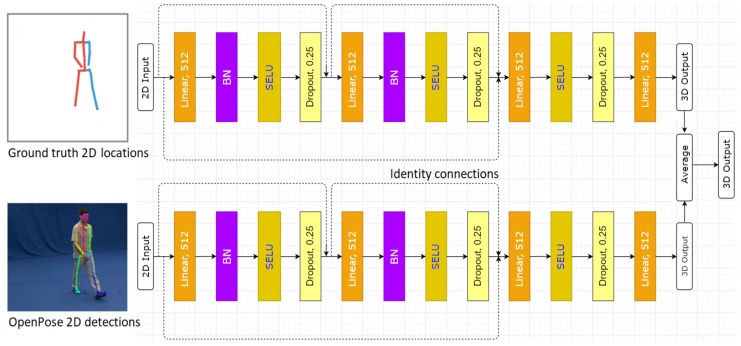
Diagram of the proposed two-stream network for training the 3D pose estimator.

State-of-the-art deep learning architectures such as ResNet [32], Inception-ResNet-v2 [46], DenseNet [47], or NASNet [48] have achieved an impressive performance in supervised learning tasks with high-dimensional data, e.g., 2D or 3D images. However, the use of these architectures [32,46,47,48] on low-dimensional data like the coordinates of the 2D human joints could lead to overfitting. Therefore, the design is based on a simple and lightweight multilayer network architecture without the convolution operations. The design process exploits some recent improvements in the optimization of the modern deep learning models [32,47]. Concretely, a two-stream network is proposed. Each stream comprises linear layers, Batch Normalization (BN) [49], Dropout [50], SELU [51] and Identity connections [32]. During the training phase, the first stream takes the ground truth 2D locations as input. The 2D human joints predicted by OpenPose [12] are inputted to the second stream. The outputs of the two streams are then averaged. Figure 2 illustrates the network design. Please note that learning with the ground truth 2D locations for both of these streams could lead to a higher level of performance. However, training with the 2D OpenPose detections could improve the generalization ability of the network and makes it more robust during inference, when only the OpenPose’s 2D output is used to deal with action recognition in the wild.

### 3.3. 3D Pose-Based Action Recognition 

**Figure 3:**
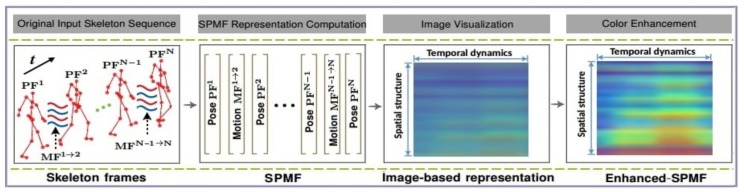
Illustration of the Enhanced-SPMF representation. To build an Enhanced-SPMF map from skeletal data, each skeleton sequence is first encoded as a single RGB image via a skeleton-based representation called SPMF (Skeleton Pose-Motion Feature) [53]. The SPMF is built from Pose Feature vectors (PFs) and Motion Feature vectors (MFs), which are calculated from the 3D coordinates of skeletons. Finally, we use a color enhancement technique [55] to enhance the local textures of the SPMF to form the Enhanced-SPMF. This is an image-based global representation for the whole input skeleton sequences. Figure reproduced, by permission from the publishers, from our previous work in [56].

This section describes the integration of the estimation stage with the recognition stage in a unified framework. Specifically, the proposed recognition approach is stacked on top of the 3D pose estimator. To explore the high-level information of the estimated 3D poses, they are encoded into a compact image-based representation. These intermediate representations are then fed to a Deep Convolutional Neural Network (D-CNNs) for learning and classifying actions. This idea has been proven effective in our previous works [52,53,54]. Thus, the spatio-temporal patterns of a 3D pose sequence are transformed into a single color image as a global representation called Enhanced-SPMF [54] via two important elements of a human movement: 3D poses and their articulation joint motions as shown in Figure 3.

**Figure 4:**
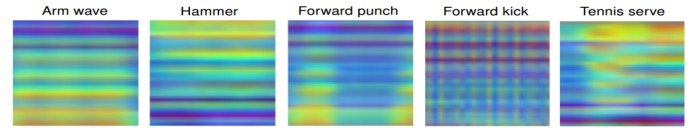
Immediate image-based representations for the recognition stage.

For a detailed technical description of the Enhanced-SPMF the interested reader is referred to the work described in [54]. Figure 4 visualizes some Enhanced-SPMF representations from samples of the MSR Action3D dataset [19].

**Figure 5:**
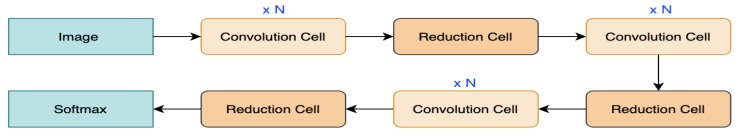
Illustration of a deep neural network generated by ENAS that contains 3 blocks, each with *N* optimal convolution cells and one reduction cell.

For learning and classifying the obtained images, the use of the Efficient Neural Architecture Search (ENAS) [14]—a recent state-of-the-art technique for automatic design of deep neural networks, is proposed. ENAS is in fact an extension of an important advance in deep learning called NAS [48], which can automate the designing process of convolutional architectures on a dataset of interest. The method searches for optimal building blocks (called cells, including normal cells and reduction cells) and the final architecture is then constructed from the best cells. Figure 5 shows a typical CNN architecture that is generated by ENAS.

**Figure 6:**
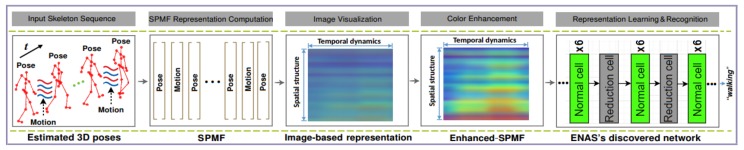
Illustration of the proposed approach for 3D pose-based action recognition.

In NAS, an RNN is used. It first samples a candidate architecture called child model. This child model is then trained to converge on the desired task and to report its performance. Next, the RNN uses the performance as a guiding signal to find a better architecture. This process is repeated many times, making NAS computationally expensive and time-consuming (e.g., on CIFAR-10, NAS needs 4 days with 450 GPUs to discover the best architecture). The main limitation of NAS is that the training of each child model to convergence requires a significant amount of time and computational resources as it measures model accuracy while throwing away all the trained weights. Therefore, ENAS has been proposed to improve the efficiency of NAS. Its key idea [14] is the use of shared parameters among child models, which helps reducing the training times of each child model from scratch to convergence. State-of-the-art performance has been achieved by ENAS on well-known public datasets. We encourage the readers to refer to the original paper [14] for more details. Figure 6 illustrates the entire pipeline of our approach for the recognition stage.

## 4. Experiments

### 4.1. Datasets and Settings

The proposed method is evaluated on three challenging datasets: Human3.6M, MSR Action3D and SBU Kinect Interaction. Human3.6M is used for evaluating 3D pose estimation. Meanwhile, the other two datasets are used for evaluating action recognition. The characteristics of each dataset are as follows.

**Human3.6M** [18]: This is a very large-scale dataset containing 3.6 million different 3D articulated poses captured from 11 actors for 17 actions, under 4 different viewpoints. For each subject, the dataset provides 32 body joints, from which only 17 joints are used for training and computing scores. In particular, 2D joint locations and 3D poses ground truth are available for evaluating supervised learning models.

**MSR Action3D** [19]: This dataset contains 20 actions, performed by 10 subjects. Experiment were conducted on 557 video sequences of the MSR Action3D, in which the whole dataset is divided into three subsets: AS1, AS2, and AS3. There are 8 actions classes for each subset. Half of the data is selected for training and the rest is used for testing.

**SBU Kinect Interaction** [20]: This dataset contains a total of 300 interactions, performed by 7 participants for 8 actions. This is a challenging dataset as it contains pairs of actions that are difficult to distinguish such as “exchanging objects–shaking hands” or “pushing–punching”. The dataset is randomly split into 5 folds, in which 4 folds are used for training and the remaining 1 fold is used for testing.

### 4.2. Implementation Details

The proposed networks were implemented in Python with Keras/TensorFlow backend. The two streams of the 3D pose estimator are trained separately with the same hyperparameters setting, in which mini batches of 128 poses are used with 0.25 dropout rate. The weights are initialized with He initialization [57]. Adam optimizer [58] is used with default parameters. The initial learning rate is set to 0.001 and is decreased by a factor of 0.5 after every 50 epochs. The network is trained for 300 epochs from scratch on the Human3.6M dataset [18]. For action recognition task, OpenPose is run [12] to generate 2D detections on MSR Action3D [19] and SBU Kinect Interaction [20]. The 3D pose estimator pre-trained on Human3.6M [18] is then used to provide 3D poses. Standard data pre-processing and augmentation techniques are used, such as randomly cropping and flipping on these two datasets due to their small sizes. To discover optimal recognition networks, ENAS [14] is used with the same parameter setting as the original work. Concretely, the shared parameters ω are trained with Nesterov’s accelerated gradient descent [59] using Cosine learning rate [60]. The candidate architectures are initialized by He initialization [57] and trained by Adam optimizer [58] with a learning rate of 0.00035. Additionally, each search is run for 200 epochs.

### 4.3. Experimental Results and Comparison

#### 4.3.1. Evaluation on 3D Pose Estimation

**Figure 7:**
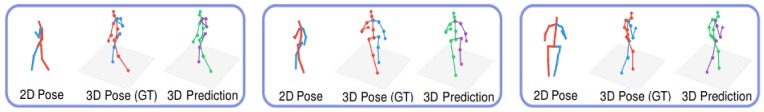
Visualization of 3D output of the estimation stage with some samples on the test set of Human3.6M [18]. For each example, from left to right are 2D poses, 3D ground truths and the 3D predictions, respectively.

The effectiveness of the proposed 3D pose estimation network is evaluated using the standard protocol of the Human3.6M dataset [18,27,29,34]. Five subjects S1, S5, S6, S7, S8 are used for training and the remaining two subjects S9, S11 are used for evaluation. Experimental results are reported by the average error in millimeters between the ground truth and the corresponding predictions over all joints. Much to our surprise, this method outperforms the previous best result from the literature [34] by 3.1mm, corresponding to an error reduction of 6.8% even when combining the ground truth 2D locations with the 2D OpenPose detections. This result proves that the network design can learn to recover the 3D pose from the 2D joint locations with a remarkably low error rate, which to the best of our knowledge, has established a new state-of-the-art on 3D human pose estimation (see Table 1 and Figure 7).

#### 4.3.2. Evaluation on Action Recognition

**Figure 8:**
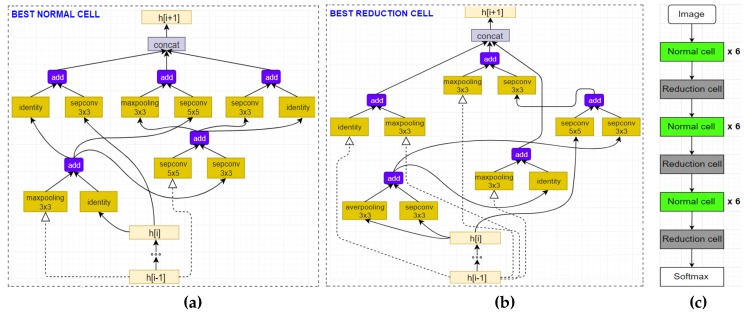
Diagram of the top performing normal cell (**a**) and reduction cell (**b**) discovered by ENAS [14] on AS1 subset [19]. They were then used to construct the final network architecture (**c**). We recommend the interested readers to see [14] to better understand this procedure.

Table 2 reports the experimental results and comparisons with state-of-the-art methods on the MSR Action3D dataset [19]. The ENAS algorithm [14] is able to explore a diversity of network architectures and the best design is identified based on its validation score. Thus, the final architecture achieved a total average accuracy of 97.98% over three subset AS1, AS2 and AS3. This result outperforms many previous studies [9,19,37,38,67,68,69,70,71], and among them, many are depth sensor-based approaches. Figure 8 provides a schematic diagram of the best cells and optimal architecture found by ENAS on the AS1 subset [19]. For the SBU Kinect Interaction dataset [20], the best model achieved an accuracy of 96.30%, as shown in Table 3. The results reported here indicated an important observation that by using only the 3D predicted poses, it was possible to outperform previous works reported in [37,72,73,74,75,76,77] and reach state-of-the-art results provided in [54,78], which deploy accurate skeletal data provided by Kinect v2 sensor.

### 4.4. Computational Efficiency Evaluation

On a single GeForce GTX 1080Ti GPU with 11GB memory, the runtime of OpenPose [12] is less than 0.1*s* per frame for an image size of 800 × 450 pixels. On the Human3.6M dataset [18], the 3D pose estimation stage takes around 15*ms* to complete a pass (forward + backward) through each stream with a mini batches of size 128. Each epoch was done within 3 min. For the action recognition stage, our implementation of the ENAS algorithm takes about 2 h to find the final architecture (∼2.3M parameters) on each subset of MSR Action3D dataset [19], while it takes around 3 h on the SBU Kinect Interaction dataset [20] to discover the best architecture (∼3M parameters). With small architecture sizes, the discovered networks require low computing time for the inference stage, making the approach more practical for large-scale problems and real-time applications.

## 5. Conclusions

In this paper, a unified deep learning framework for joint 3D human pose estimation and action recognition from RGB video sequences has been presented. The proposed method first runs a state-of-the-art 2D pose detector to estimate 2D locations of body joints from a monocular RGB sensor, although the approach is not limited to a particular 2D pose detector. A deep neural network was then designed and trained to learn a direct 2D-to-3D mapping and predict human poses in 3D space. Experimental results demonstrated that the 3D human poses can be effectively estimated by a simple network design and training methodology over 2D keypoints. A novel action recognition approach was also introduced based on a compact image-based representation and automated machine learning, in which an advanced neural architecture search algorithm was exploited to discover the best performing architecture for each recognition task. The experiments on public and challenging action recognition datasets indicated that the proposed framework was able to reach state-of-the-art performance, while requiring less computation budget for training and inference. Despite that, this method naturally depends on the quality of the output of the 2D detectors. Hence, a limitation is that it cannot recover 3D poses from 2D failed output. To tackle this problem, we are currently expanding this study by adding more visual evidence to the network to further gains in performance. The preliminary results are encouraging. Codes and models will be made available on our GitHub project at https://github.com/huyhieupham/. 

## Figures and Tables

**Table 1:** Experimental results (average error in mm) and comparison with previous state-of-the-art 3D pose estimation approaches on the Human3.6M dataset [18]. The symbol ${}^{🟉}$ denotes that a 2D detector was used and the symbol ^†^ denotes the ground truth 2D joint locations were used.

Method	Direct.	Disc.	Eat	Greet	Phone	Photo	Pose	Purch.	Sit	SitD	Smoke	Wait	WalkD	Walk	WalkT	Avg
Ionescu et al. [18]^†^	132.7	183.6	132.3	164.4	162.1	205.9	150.6	171.3	151.6	243.0	162.1	170.7	177.1	96.6	127.9	162.1
Du et al. [61]${}^{🟉}$	85.1	112.7	104.9	122.1	139.1	135.9	105.9	166.2	117.5	226.9	120.0	117.7	137.4	99.3	106.5	126.5
Tekin et al. [26]	102.4	147.2	88.8	125.3	118.0	182.7	112.4	129.2	138.9	224.9	118.4	138.8	126.3	55.1	65.8	125.0
Park et al. [62]${}^{🟉}$	100.3	116.2	90.0	116.5	115.3	149.5	117.6	106.9	137.2	190.8	105.8	125.1	131.9	62.6	96.2	117.3
Zhou et al. [63]${}^{🟉}$	87.4	109.3	87.1	103.2	116.2	143.3	106.9	99.8	124.5	199.2	107.4	118.1	114.2	79.4	97.7	113.0
Zhou et al. [64]${}^{🟉}$	91.8	102.4	96.7	98.8	113.4	125.2	90.0	93.8	132.2	159.0	107.0	94.4	126.0	79.0	99.0	107.3
Pavlakos et al. [27]	67.4	71.9	66.7	69.1	72.0	77.0	65.0	68.3	83.7	96.5	71.7	65.8	74.9	59.1	63.2	71.9
Mehta et al. [65]${}^{🟉}$	67.4	71.9	66.7	69.1	71.9	65.0	68.3	83.7	120.0	66.0	79.8	63.9	48.9	76.8	53.7	68.6
Martinez et al. [34]${}^{🟉}$	51.8	56.2	58.1	59.0	69.5	55.2	58.1	74.0	94.6	62.3	78.4	59.1	49.5	65.1	52.4	62.9
Liang et al. [66]	52.8	54.2	54.3	61.8	53.1	53.6	71.7	86.7	61.5	53.4	67.2	54.8	53.4	47.1	61.6	59.1
Luvizon et al. [44]	49.2	51.6	47.6	50.5	51.8	48.5	51.7	61.5	70.9	53.7	60.3	48.9	44.4	57.9	48.9	53.2
Martinez et al. [34]^†^	37.7	44.4	40.3	42.1	48.2	54.9	44.4	42.1	54.6	58.0	45.1	46.4	47.6	36.4	40.4	45.5
**Ours** ${}^{†,}$ ^🟉^	**36.6**	**43.2**	**38.1**	**40.8**	**44.4**	**51.8**	**43.7**	**38.4**	**50.8**	**52.0**	**42.1**	**42.2**	**44.0**	**32.3**	**35.9**	**42.4**

**Table 2:** Test accuracies (%) on the MSR Action3D dataset [19]. Please note that many previous methods are based on depth sensors.

Method	AS1	AS2	AS3	Aver.
Li et al. [19]	72.90	71.90	71.90	74.70
Chen et al. [67]	96.20	83.20	92.00	90.47
Vemulapalli et al. [9]	95.29	83.87	98.22	92.46
Du et al. [38]	99.33	94.64	95.50	94.49
Liu et al. [37]	N/A	N/A	N/A	94.80
Wang et al. [68]	93.60	95.50	95.10	94.80
Wang et al. [69]	91.50	95.60	97.30	94.80
Xu et al. [70]	99.10	92.90	96.40	96.10
Lee et al. [71]	95.24	96.43	100.0	97.22
Pham et al. [54]	98.83	99.06	99.40	99.10
**Ours**	**97.87**	**96.81**	**99.27**	**97.98**

**Table 3:** Test accuracies (%) on the SBU Kinect Interaction dataset [20]. Please note that many previous methods are based on depth sensors.

Method	Acc.
Song et al. [72]	91.51
Liu et al. [37]	93.30
Weng et al. [73]	93.30
Ke et al. [74]	93.57
Tas et al. [75]	94.36
Wang et al. [76]	94.80
Liu et al. [77]	94.90
Zang et al. [78] (using VA-RNN)	95.70
Zhang et al. [78] (using VA-CNN)	97.50
Pham et al. [54]	97.86
**Ours**	**96.30**
